# Palliative Care: Taking the Long View

**DOI:** 10.3389/fphar.2018.01140

**Published:** 2018-10-16

**Authors:** María Teresa García-Baquero Merino

**Affiliations:** Facultad de Medicina, Universidad Católica San Antonio de Murcia, Murcia, Spain

**Keywords:** palliative medicine, ethos, barriers, medication, epidemiology, ethics, policy

## Abstract

Our medicalised modern cultures render reason and mystery mutually exclusive, define death by disease as failure, and dying as disgraceful. Providers and policymakers alike marginalize aging and dying individuals, formulating largely ineffective strategies without palliative care and pain relief budgets. The aim of palliative care is to support the person with incurable illness to live their remaining life as well and as meaningfully as possible and to support them as they eventually die from their illness and reaching the natural end of their lives It acknowledges that each life is morally significant, restoring patients' and families' quality of life where possible, and attending meticulously to the dying period as necessary (Saunders, 1965). Hospices are far more than mere buildings; they house an ethos of care. The field is currently challenged by its variable situation over the world and the pressing need to incorporate new technology to its practice. This article provides a review of some important milestones in the history and development of Palliative Care and evolution of Palliative Medicine in some countries, some current issues concerning consistency in its implementation, and some likely prospects for its future advance and expected expansion, from the perspective of one central question: “What constitutes the ethos of Palliative Care replicating its foundational philosophy and principles?” which helps to set the scene for possible future advances to integrate ethical, legal, and social implications. Technology will help expansion by facilitating communication and predicting needs.

## Introduction

Death as an expected part of life, is a truism acknowledged by all and fought by many. Before death, there is often dying as a process that entails effort and response which Western society (Zimmermann and Rodin, 2004) as a whole seems to fight shamelessly, led by health systems inherently enthralled with extending life beyond what current scientific evidence predicts; consequently, life natural limits are blurred. Simultaneously, people in many regions (Deutsche Gesellschaft für Palliativmedizin, 2014) have started a senseless dance with the right to curtail lives in order to limit the anguish their citizens endure when their life is no longer deemed to be worth living. Professional associations positioning statements certainly help sharpen conceptual limits but, is this enough to balance the enormous amount of confusing and contradictory existing information by non-specialists which themselves don't seem to grasp the idiosyncrasies of palliating suffering respecting life and valuing it as it is while accepting death without bringing it about or delaying it? (Coulter, 2017) It might not be the case

Palliative Care enacts new patterns and algorithms that signify an evolutionary turn. “Evolution…becomes primarily a psychosocial process, based on the cumulative transmission of experience and results, and working through an organized system of awareness, a combined operation of knowing, feeling, and willing which gives rise to new patterns of cooperation, new organizations of awareness, new and often wholly unexpected possibilities have been realized,” wrote Sir Julian Huxley (Teillhard de Chardin, 1963). Palliative care is one such new pattern needing to be ingrained in our societies.

## The past: standing on giants' shoulders

### Some historical facts. advances

The twentieth century saw Dame Cicely Saunders bring about the modern hospice movement (Lutz, 2011), in the U.K., born from her profound understanding and need to respond to the suffering she witnessed in London's hospitals where the focus of care was shifting away from the needs of who could no longer benefit from better technology and lifesaving treatment. Palliative care has its modern roots in her concern regarding the poor care being provided to dying hospital patients. Descendant of the medieval pilgrimages, the Modern Hospice Movement was thus born to use scientific rigor to research and treat symptoms such as pain, with its focus set holistically on the physical, emotional, social, and spiritual needs of patients.

“The longer I work with the dying and their families the more I learn about life and the small things that are so important to each one of us. Dying is never easy,” Cicely stated (Saunders, 1965).

The word Palliative is derived from the Latin word “pallium” meaning to cloak: symptoms are eased while the underlying condition cannot be cured. Wrapping the person and that which matters to her in perceptible care and understanding helps them be master of one's life as death approaches. The philosophy of palliative care is about maximizing a person's quality of life by effective symptom control, psychological and spiritual support, in a socially meaningful way, while truly allowing someone to be themselves at a difficult time. This demands a multi-disciplinary attitude (Doyle, 1993) from which the skills of various professionals are pooled and utilized. A variety of models exist, only as good as their individual's centeredness.

Palliative care is linked synonymously with the hospice movement which began at St Christopher's Hospice in London in 1967; both are widely understood as a joint approach nowadays provided by many healthcare settings to some degree. Hospices having proliferated unevenly, dependent on cultural heritage; far more than mere buildings, they house an ethos of care behind which sits a highly compassionate all-inclusive approach placing the wellbeing of patients and their families as its very core.

## The present: a kaleidoscope of barriers, challenges and opportunities

The WHO (2018) defines palliative care as an “approach that improves the quality of life of patients and their families facing the problems associated with life-threatening illness, through the prevention and relief of suffering by means of early identification and impeccable assessment and treatment of pain and other problems, physical, psychosocial and spiritual.” This means all treatment and care options available for people with incurable, life-threatening conditions and ages, wherever in the world they are and emphasizes a special interdisciplinary and multi-professional character. It lists a series of helpful categorical pronouncements that clarify what palliative care is or does, isn't or doesn't. Its latest version didn't refer to patient values, wishes, or choice, which means WHO guides professionals but not society in general or particular patients. The risk is that if professionals don't know what patients want, they may end up providing them with what they wouldn't have chosen which might compromise their wellbeing.

### Current difficulties distinguishing palliative care from the specialty of palliative medicine

Palliative care has evolved, in some countries, as a medical specialty-−1987 in United Kingdom; 2006 in the United States—to be one of the latest fields of medical expertise, known as Palliative Medicine (GMC, 2010).

Much thought went into the very first definition of Palliative medicine in 1987 in the U.K. Agreed as “The study and management of patients with active, progressive, far-advanced disease for whom the prognosis is limited and the focus of care is the quality of life” (Doyle, 1993). Its rich content making timeless: focused on quality rather than quantity of life, didn't limit its care to any single pathology nor did it impose time limit. It did exclude those with chronic conditions which, in themselves, don't imminently threaten life. It was soon widely accepted and adapted by others.

Derek Doyle addressed the confusion created by that first definition as it related to the stage of a patient's condition, rather than its pathology, body system or a particular technical approach to its management. Doyle emphatically argued in favor of a concerted and coordinated effort by the palliative medicine specialists to somehow “bring it of age,” and set its scene to resemble it to that of other medical specialities. Such an effort would address the need to delimitate: palliative care approach; general palliative care; hospice; specialist palliative care unit; palliative medicine and supportive care, currently used in an improvised way.

The Specialty Training Curriculum for Palliative Care UK Syllabus, developed under the direction of the Joint Royal Colleges of Physicians Training Board and in line with General Medicine College, deftly deals with the medical challenge by defining The Palliative Medicine Speciality as “the branch of medicine involved in the treatment of patients with advanced, progressive, life-threatening disease for whom the focus of care is to optimize their quality of life through expert symptom management an psychological, social and spiritual support as part of a multi professional team,” going on to insist that “Palliative Medicine specialists may work in hospital, in the community and hospices or other specialist palliative care units.”

Well into the twenty-first century, medical technology has afforded advances in absolutely every one of the healthcare fields improving health, prolonging life. These advances can make society struggle to humanize or personalize care; health and social professionals find ever- increasing difficulties to facilitate patient's autonomous decision-making. Compassionate care does not flow; a deep and wide urge to ensure patient values and choice about their own health and social matters is gaining momentum contributing to the rapid development experienced by Palliative Care (Clark, 1993). It has been unlike that of almost any other area of healthcare; it has received considerable socio- political support due to the epidemiological developments expected in our society (Barclay et al., 1997). The consistent focus it affords to the needs of patients and their families in such an existential situation has led to its provision growing in the developed world though much less so in poorer ones over the last decade (Clark et al., 1997). International organizations, scientific associations, publish documents guiding adherence to adequate standards (Hillier, 1988). Professionals, teams and services are increasingly available; paradoxically, gaps in population access remain disturbingly widespread, a fact that needs to be addressed (McNamara et al., 1994).

As we celebrate the Centenary of the birth of Cicely—nurse, almoner, doctor, founder, humanist, carer, and patient as she so liked to remind those close to her—only a handful of countries has adopted Palliative Care philosophy and principles formally in 2018 (Higginson, 1993). Why might that be?

### The published state of the situation

The literature confirms urgent global need for palliative care, determination of many to provide services while highlighting the real implementation effort undertaken from international bodies all the way down to other specialties (Gilbert, 1996). More than 75% of the countries of the world do not provide palliative care though many are determined to address the issue (Centeno et al., 2007).

The Oxford Textbook of Palliative Medicine (Cherny et al., 2015) chapter “Barriers to deliver palliative care” in which it reviews its definition, considering the concept within. A concept not fully understood but “generally embraced throughout the world.” It recognizes the lack of implementation in routine clinical care, recognizing blocking factors including difficulties of distinguishing between generalist and specialist care. It acknowledges palliative care as human right, without establishing qualitative or quantitative criteria or duties to honor that right! It suggests the WHO as the crucial organization to develop palliative care implementation.

The WHO, struggles to find minimum criteria to apply throughout and uses morphine consumption for reported cancer deaths as an indicator of palliative care availability (WHO, 2018), while in some countries, the use of prescribed Fentanyl—many times the price of the morphine—has seen its prescribing incremented.

The WPCA global Atlas of Hospice and Palliative Care Development (WHO, 2014) identifies two major barriers to palliative care implementation: health care education and access to essential palliative care medications.

Different reports highlight the particular challenges present in poorer countries such as the HIV/AIDS epidemic in Sub-Saharan Africa (Harding et al., 2006). In only 33 of 80 countries assessed in a large study are opioid analgesics freely available to prescribe; red tape and legal restrictions commonly obstructing symptom control.

China's specialists (Hawley, 2017), reflect on the main barriers found in a region of rapid population growth and aging society: palliative care is considered to be just for the dying; financial implications and the absence of national strategies; and severe shortage of trained health professionals. Others point at clinical challenges, such as not having the “know-how” to respond to clinically complex situations as barriers.

Professor I Findlay's (Finlay, 2006) outlines a range of barriers in the UK, including: GP and Community working patterns, lack of continuity of care, Failure to respond at time of need, Differencing between generalist, specialist need and being able to shoulder the burden, attitudes sacrificed in the political drive for targets, too simple outcomes and financial expediency, diseases other than cancer huge unmet needs, aged population, lack of knowledgeable, skilled staff, 24 h palliative care services and training. Problems with terminology and conceptual clarity, are latent in all research papers, where mixing of palliative care benefits, barriers and best practices are too common. These issues are beginning to be explicitly addressed by different authors, appearing in EAPC Conference proceedings too.

International specialists such as Anne Merriman talk of the importance of upholding the hospice ethos—the hospice as host and the patient as guest—and the spiritual aspects and principles behind palliative care, the problems and challenges the hospice movement faces and, ultimately, the importance of the special calling of working with the dying. Others ponder on how intangible values are expressed in concrete actions which transmit unconditional acceptance and affirm patient's inner worth as a person.

### The role of technology

It is important to consider the possible impact technology might have in helping improve quality of life more widely. Researchers and clinicians are finding ways to harness IT to facilitate communication between patients and caregivers. Telemedicine contributes to facilitate independence (Avati et al., 2018) monitoring patients remotely: patients and their families can communicate in real time with their palliative care team, wherever their respective locations. Videoconferences are allowing professionals to provide support and advice to others and its use for multidisciplinary team improve the, always elusive, continuity of care.

“Simple technological interventions have an impact on access,” says C Ritchie, “as well as helping with a major problem: the shortage of trained specialists, particularly in rural areas, along with the insufficient training of generalist care providers.” Getting to a clinic can be burdensome for seriously ill patients living far from the nearest palliative care specialist. Research shows the way to new ways of being present and listening actively: a randomized, controlled trial showed participants in the ENABLE intervention had better scores for quality of life, mood, and symptom intensity, even improved survival when face-to-face access to services can be difficult. Technology will never take the place of presence and engaging therapeutic interactions, but benefits of some contact outweigh the potential burdens of no contact.

## The future: outgrowth of palliative care, keeping the hospice movement ethos

It might be tempting to feel overwhelmed by the many challenges besieging this field or give in to the ever-present risk of oversimplifying a complex reality. To make noteworthy the strength of the field we should work on well-established foundations to support the deepest certainties which allow people to make sense of their lives while facing impending death.

It is while working within the ground rules that frame the worth of the most fragile lives and the inner strengths of the most vulnerable, that professionals acquire understanding about the natural ending of life, the small things that are important and meaningful to the dying individual. Then to become a resource for the community, showing leadership in care and collaboration.

Support from comprehensive policies and legislative clarity are called for Twycross (2002). The current dearth of regional and national policies within Public Health populational-sensitive frameworks might be leading to an increase in legislation, the appropriateness of which is, at best questionable, mostly counter-productive regarding to palliative care development. Policy takes in whole populations; laws aim to protect the weaker members of a population in relation to a particular issue that might be imposed on them by members in stronger positions. Legislation can lead to segregation and increasing vulnerability, the opposite of what palliative care came into being to put an end to.

Palliative Care has a clear remit not to be confused with other approaches claiming that the outcome, death, is the same and that the intention of both is to relieve suffering. These assertions are wrong: there is a moral distinction between giving a lethal injection and the interventions that constitute Palliative Care.

National and international recommendations (ESMO, 2012) and policies increasingly call all providers involved to identify the beginning of irreversible processes, facilitate decision-making in practice and care for those with life-threatening conditions, with the vast majority of the existing body of scientific evidence on rigorous and assertive symptom control and management coming from Oncology (Rabow et al., 2015). Much remains to be done as lack of palliative care in more than 80% of the world, and lack of the palliative care medicines containing “narcotic drugs,” has driven what experts call the “global pandemic of untreated pain.”

A palliative culture would halt and slowly reverse this pandemic, whose source is cumulative indifference and ignorance dating back decades.

There are multiple concerns to the involvement of palliative care coming from other disciplines, primarily amongst those are apprehensions by physicians that a referral to palliative care is appropriate only when all active treatment options are exhausted, that such a referral will “send the wrong signal” or “take away their hope.” These barriers may be precisely the same ones preventing clinicians having an open and honest discussion with the patient about prognosis, quality of life and the limitations to treatment. Realism is suspended by a ribbon of hope. If such hope is singularly invested in cure or remission then when that becomes impossible, great disappointment may ensue.

Year on year, advances can be observed and systematically developed therapeutic recommendations made available on the basis of best scientific evidence and clinical experience of ever-growing numbers of local and regionally sensitive champions. These champions' influence can be challenging because the fantastic impulse they afford the field can also blur its boundaries. Bringing new aspects of disease and specialist level-specific knowledge and experience, incorporating new “know-how” and recommendations to agree adjustments to reflect the varying situations can also help palliative care consolidate its Lex Artis.

## Conclusion

As a society, we are drowning in information but starving for the wisdom required for making clinical care more effective, efficient and agreeable by getting care as good as it can be wherever the person is—at all stages—care that matches the person's preferences as closely as possible and meet needs as far as possible.

Last year was the 30th anniversary of the Palliative Medicine being recognized as a medical specialty. Much has been achieved, but there is still much that needs to be done to ensure good palliative care (and supportive/end-of-life care) for everyone. Equally, we face new challenges, especially relating to service provision and staff need appropriate training and specialist training too have confidence to take their skills into other parts of care—i.e., further upstream and laterally to start reducing the inequality gap.

Research shows that those who die at home experience more peace and a similar amount of pain than those who die in hospital and their relatives also experience less grief. Too much emphasis might be put on the place of death; home might not always be the best or preferred place of death. Public Health focus on place of death distracts attention from the experience of dying and deflects attention from many more pressing factors that limit patients' options, such as availability of resources, quality of palliative care, and symptom management.

We must be aware of the projected increase in institutional deaths; the hospital needs to be reinvented as a viable alternative and place of excellent care for dying patients and their families. How to deliver consistently compassionate and effective support for dying people and their families in all settings remains a crucial challenge while caring for a growing aging Western population. But “Disadvantaged dying” (Saunders, 1965)—everyone who can't get access to good end of life care must be taken care of too both in the developed and in the poorer countries.

Strong social networks facilitate palliation and everybody feeling responsible for playing a positive part in end of life care will end up showing care to be about return on humanity, not return on investment.

## Author contributions

The author confirms being the sole contributor of this work and has approved it for publication.

### Conflict of interest statement

The author declares that the research was conducted in the absence of any commercial or financial relationships that could be construed as a potential conflict of interest.
